# Comparing severity between *Plasmodium falciparum* malaria and sepsis

**DOI:** 10.1186/1475-2875-9-S2-P10

**Published:** 2010-10-20

**Authors:** Ermelindo M Filipe, Lina Antunes, Esmael F Tomás, Edna Viegas, João Bernardo, Fortunato R Silva

**Affiliations:** 1 Sagrada Esperança Clinic ICU resident; 2 Sagrada Esperança Clinic ICU specialist

## Introduction

Malaria is the most important human parasitic disease, affecting about 40% worldwide population, most prevalent in tropical countries. Severe *Plasmodium Falciparum* Malaria is a cause of ICU in coming, appearing like in Sepsis with multi - organ failure, resulting in high mortality 10-50%. Non-immune patient are most susceptible.

## Objectives

Compare severity between severe *Plasmodium falciparum* Malaria and Sepsis.

## Method

Severity between the 18 patients admitted with Severe *Plamodium falciparum* Malaria and the first 18 patients admitted with Sepsis in ICU -Sagrada Esperança Clinic during 2007 was compared concerning to age, sex, severity ICU scores (SOFA and APACHE II), organ failure and mortality. We also compared mortality between immune and non-immune patients for Malaria.

## Results

See tables 1 and 2

**Table 1:** Comparing severity

	Malaria n=18	Sepsls n=18	*P*
Age (X±SD)	39±12	41±13.24	ns
Sex	M: 61%	M: 64%	
SOFA admission (X)	17	18	ns
SOFA 3rd Day(X)	19	15	ns
SOFA 7th Day(X)	17	9	ns
Apache II	10	12	ns
Mortality	27.8%	47.4%	

**Table 2:** Mortality under immunity for Malaria

	Immune n = 14	Non-immune n=4
Mortality	17%	**50%**

**Table 3:** Organ failure

	Malaria n=18	Sepsis n=18
Neurologic	11.1%	16.7%
Respiratory	0%	16.7%
Hematologic	33.3%	3%
Hepatic	44.4%	33.3%
Hemodynamic	5.6%	27.7%
Renal	33%	33.3%

## Conclusion

There is no significant difference for severity comparing severe *plasmodium falciparum* malaria and Sepsis, there are similar organ failure rate.

Mortality among non-immune patients is high.
